# Transition From Proto-Kranz-Type Photosynthesis to HCO_3_^–^ Use Photosynthesis in the Amphibious Plant *Hygrophila polysperma*

**DOI:** 10.3389/fpls.2021.675507

**Published:** 2021-06-16

**Authors:** Genki Horiguchi, Kaori Matsumoto, Kyosuke Nemoto, Mayu Inokuchi, Naoki Hirotsu

**Affiliations:** ^1^Graduate School of Life Sciences, Toyo University, Gunma, Japan; ^2^Faculty of Life Sciences, Toyo University, Gunma, Japan; ^3^Department of Aquatic Bioscience, Graduate School of Agricultural and Life Sciences, The University of Tokyo, Tokyo, Japan

**Keywords:** bicarbonate use, proto-Kranz anatomy, carbon concentrating mechanism, amphibious plant, Acanthaceae, submergence

## Abstract

*Hygrophila polysperma* is a heterophyllous amphibious plant. The growth of *H. polysperma* in submerged conditions is challenging due to the low CO_2_ environment, increased resistance to gas diffusion, and bicarbonate ion (HCO_3_^–^) being the dominant dissolved inorganic carbon source. The submerged leaves of *H. polysperma* have significantly higher rates of underwater photosynthesis compared with the terrestrial leaves. 4,4′-Diisothiocyanatostilbene-2,2′-disulfonate (DIDS), an anion exchanger protein inhibitor, and ethoxyzolamide (EZ), an inhibitor of internal carbonic anhydrase, repressed underwater photosynthesis by the submerged leaves. These results suggested that *H. polysperma* acclimates to the submerged condition by using HCO_3_**^–^** for photosynthesis. *H. polysperma* transports HCO_3_**^–^** into the leaf by a DIDS-sensitive HCO_3_**^–^** transporter and converted to CO_2_ by carbonic anhydrase. Additionally, proteome analysis revealed that submerged leaves accumulated fewer proteins associated with C4 photosynthesis compared with terrestrial leaves. This finding suggested that *H. polysperma* is capable of C4 and C3 photosynthesis in the terrestrial and submerged leaves, respectively. The ratio of phosphoenol pyruvate carboxylase to ribulose 1,5-bisphosphate carboxylase/oxygenase (Rubisco) in the submerged leaves was less than that in the terrestrial leaves. Upon anatomical observation, the terrestrial leaves exhibited a phenotype similar to the Kranz anatomy found among C4 plants; however, chloroplasts in the bundle sheath cells were not located adjacent to the vascular bundles, and the typical Kranz anatomy was absent in submerged leaves. These results suggest that *H. polysperma* performs proto-Kranz type photosynthesis in a terrestrial environment and shifts from a proto-Kranz type in terrestrial leaves to a HCO_3_**^–^** use photosynthesis in the submerged environments.

## Introduction

Carbon assimilation is essential for plant growth. In stress conditions emanating from high temperature, high light intensity, salt (Chen et al., 2015), or drought (Flexas and Medrano, 2002), stomata are closed to suppress water loss, causing decreases in gas exchange and increases in photorespiration. Higher plants evolved C4 photosynthesis and crassulacean acid metabolism (CAM) to maintain carbon assimilation rates under conditions limiting carbon acquisition. C4 photosynthesis and CAM concentrate CO_2_ around ribulose 1,5-bisphosphate carboxylase/oxygenase (Rubisco) to avoid limiting carbon assimilation condition such as when the stomata are closed. C4 photosynthesis concentrates CO_2_ by physically separating primary CO_2_ fixation by phosphoenol pyruvate carboxylase (PEPC) in mesophyll cells (MCs) from secondary fixation by Rubisco in bundle sheath cells (BSCs) (Hatch, 1971; Kanai and Edwards, 1973). In contrast, CAM plants fix CO_2_ using PEPC during the night and conduct secondary fixation by Rubisco during the day (Black et al., 2003). C4 photosynthesis and CAM are adaptations allowing photosynthesis to continue in limiting CO_2_ conditions (Sage et al., 2012).

C4 photosynthesis and CAM are found not only in land plants but also in aquatic plants (Yin et al., 2017). In submerged conditions, gas diffusion is 10^4^ times slower compared with the terrestrial condition, thereby limiting dissolved inorganic carbon (DIC) and oxygen availability (Maberly and Madsen, 2002). Moreover, the relative distribution of DIC constituents (CO_2_, HCO_3_^–^, and CO_3_^2–^) depends mainly on the pH of the aqueous environment (Pedersen et al., 2013). Bicarbonate ion (HCO_3_^–^) is the dominant DIC form between pH 7.0 and pH 10.0. Most freshwater lakes have a pH value that range from 6 to 8 (Hasler et al., 2018), HCO_3_^–^ is the dominant DIC form in nature. Submerged environments usually have low CO_2_ levels; thus, C4 photosynthesis and CAM have the advantage. Some aquatic plants switch photosynthetic pathways with changing external environments. *Egeria densa* and *Hydrilla verticillata* (Hydrocharitaceae), aquatic monocots, typically perform C3 photosynthesis but induce single-cell C4 photosynthesis under carbon-limiting conditions (Casati et al., 2000; Bowes et al., 2002). *Ottelia alismoides* (Hydrocharitaceae), a freshwater monocot, exhibits C4 characteristics (Zhang et al., 2014; Shao et al., 2017; Han et al., 2020) and increases CAM activity under low CO_2_ conditions (Zhang et al., 2014). *Isoetes howellii* and *I. sinensis* (Isoetaceae), amphibious lycophytes, increase CAM activity in leaves that develop in the submerged condition but not in leaves that develop in the terrestrial condition (Keeley, 1983; Yang and Liu, 2015). Leaves with CAM activity have a higher net photosynthetic rate and lower apparent photorespiration in the submerged environment (Pedersen et al., 2011). *Eleocharis* species, amphibious monocots, use diverse photosynthetic carbon assimilation pathways (C3, C3–C4, C4–like, and C4) in their terrestrial and submerged leaves (Murphy et al., 2007). Interestingly, *Eleocharis* species are unlike other plants that can switch their photosynthetic metabolism; the terrestrial leaves of *Eleocharis vivipara* exhibit C4 characteristics, whereas submerged leaves exhibit C3 characteristics (Ueno et al., 1988; Murphy et al., 2007). The alteration of photosynthetic type in *E. vivipara* is confirmed not only in enzymatic localization and activities but also in anatomical structure as the Kranz anatomy is absent in submerged leaves.

Leaf anatomy reflects the photosynthetic capacities for both CO_2_ and light. To concentrate CO_2_ in BSCs, larger BSCs are needed that contain more abundant organelles (Lundgren, 2020). Aquatic plants typically have reduced leaf thicknesses, cuticles, and stomatal densities compared with terrestrial plants (Veen and Sasidharan, 2019). Leaves that are thin or have a thin cuticle have lower gas diffusion resistance properties (Frost-Christensen et al., 2003; Mommer et al., 2005). The presence of chloroplasts in epidermal cells is a common feature in aquatic plants (Rascio, 2002; Borum et al., 2016; Han et al., 2020). Differences in cell shape and in the chloroplast content of the two mesophyll cell types, palisade and spongy tissues, are important factors in allocating light energy within leaves (Terashima and Saeki, 1983). Leaf anatomical features, especially chloroplast positioning, can influence the internal light environment and light use efficiency (Xiao et al., 2016).

Aquatic phototrophs have carbon-concentrating mechanisms (CCMs) to acclimate to low CO_2_ conditions. Algal transport of HCO_3_^–^ from the external environment to carboxysomes or pyrenoids relies on transporters such as BCT1 (Omata et al., 1999), SbtA (Shibata et al., 2002), BicA (Price et al., 2004), HLA3 (Yamano et al., 2015), and SLC4 (Nakajima et al., 2013), or channels such as LCIA (Yamano et al., 2015). Carboxysomes contain multiple forms of carbonic anhydrase (CA), including CcaA and CcmM, whereas pyrenoids contain theta CA (Duanmu et al., 2009; Kikutani et al., 2016; Gee and Niyogi, 2017), and Rubisco, where HCO_3_^–^ is converted to CO_2_. Algal CCMs are referred to as biophysical CCMs, whereas C4 photosynthesis and CAM are referred to as biochemical CCMs. Having biophysical or biochemical CCMs to increase CO_2_ availability is advantageous for submerged plants. In higher plants, HCO_3_^–^ utilization mechanisms likely include biophysical CCMs. *Potamogeton lucens* and *Elodea canadensis* exhibit a light-induced polar pH reaction (Prins et al., 1979; Elzenga and Prins, 1989). In these plants, HCO_3_**^–^** is taken and converted to CO_2_ in the lower side, and OH^–^ is excreted from the upper side. *Posidonia oceanica*, a seagrass, transports HCO_3_**^–^** into the cell by a HCO_3_^–^/H^+^ symporter (Rubio et al., 2017). The submerged leaves of *Hygrophila difformis*, an amphibious eudicot, have a HCO_3_^–^ utilization mechanism that is hypothesized to transport HCO_3_^–^ across the plasma membrane by a means other than H^+^ symport (Horiguchi et al., 2019). *O. alismoides* has two pathways, passive diffusion of CO_2_ that is converted to HCO_3_^–^ using an external α-CA and transport of HCO_3_^–^ into cells by SLC4 (Huang et al., 2020).

Higher plants have diverse acclimation strategies for low CO_2_ environments, including submergence; however, the photosynthetic acclimation strategies used by submerged plants remain unclear in eudicots. In this study, we investigated how the heterophyllous amphibious eudicot *Hygrophila polysperma* acclimated to low CO_2_ availability in a submerged condition by comparing the properties of terrestrial and submerged leaves. We hypothesized that *H. polysperma* uses not only CO_2_ but also HCO_3_^–^ as a photosynthetic carbon source and/or increases CO_2_ availability by morphological and functional change.

## Materials and Methods

### Plant Growth Conditions

Clonal seedling cuttings of *H. polysperma*, a heterophyllous amphibious plant, were used. Three plants were planted into each vinyl pot (220 ml) containing soil (Leaf Pro Soil normal, Leaf Corporation, Gunma, Japan). Ten pots were placed into each of two glass tanks (W30 cm × D30 cm × H40 cm). The pots were partially submerged in tap water such that the water level was kept below the soil surface. Plants were grown at 25°C and illuminated with a 150-W metal halide lamp (Funnel 2 8000K, Kamihata, Hyogo, Japan) with a photosynthetic photon flux density (PPFD) of 200 μmol m^–2^ s^–1^, 8-h light and 16-h dark. After 2 weeks of initial growth, plants were separated into two environmental treatments: terrestrial and submerged. The terrestrial treatment maintained the initial growing condition. The submerged treatments were achieved by adding 25 L of tap water to the tank and maintaining the same light and temperature conditions as described for the terrestrial treatment. The temperature, pH, and alkalinity in the submerged treatment at day time were 25°C, 7.38, and 0.3 mEq L^–1^, respectively. Leaves that developed after the terrestrial and submerged treatments were regarded as terrestrial and submerged leaves, respectively. The uppermost fully expanded leaves from both terrestrial and submerged treatments were used for subsequent experiments.

### Underwater Photosynthesis Measurement

Underwater oxygen evolution was monitored using a liquid phase oxygen electrode (OXYG-1, Hansatech, Norfolk, United Kingdom). Four leaf disks (6-mm diameter, total projected area of 113 mm^2^) were placed in an oxygen electrode chamber (DW1/AD, Hansatech, Norfolk, United Kingdom) containing measurement buffer whose composition was modified for every experiment. The temperature inside the oxygen electrode chamber was maintained at 25°C by submerging the chamber in a low-temperature water bath (NCB-1,200, EYELA, Tokyo, Japan). The DIC response curve for the net oxygen evolution rate was obtained for NaHCO_3_ values between 10 and 1,650 μM. In addition to NaHCO_3_, the measurement buffer contained 0.1 mM phosphate buffer (pH 6.3), 1.5 mM KCl, 1.0 mM NaCl. Photosynthesis was started by illumination at an irradiance of 285 μmol photons m^–2^ s^–1^ after dark acclimation for over 15 min. The DIC concentration increased immediately after injecting NaHCO_3_. The light response curve of the net oxygen evolution rate was measured for PPFD values from 0 to 820 μmol photons m^–2^ s^–1^ in the presence of 10 mM NaHCO_3_. The initial slopes were calculated in low DIC (10–150 μM NaHCO_3_) and light (0–125 μmol photons m^–2^ s^–1^) regions. After measuring the underwater photosynthetic rate, leaves were collected to analyze the chlorophyll content.

The leaf chlorophyll concentration was determined as described previously (Porra et al., 1989). The leaf disks for measuring underwater photosynthesis were incubated in N,N-dimethylformamide at 4°C in the dark overnight. The absorbance of the extract was measured at three wavelengths: 750.0, 663.8, and 646.8 nm. The chlorophyll concentration and content per unit leaf area were calculated from the absorbance readings.

### Measurement of HCO_3_^–^ Use

To investigate the DIC form used for photosynthesis, the photosynthetic oxygen evolution rate was measured at pH 6.3 and 8.3. DIC constituents (CO_2_ and HCO_3_^–^) present in the media at pH 6.3 or 8.3 existed as a level of 1:1 or HCO_3_^–^ only, respectively. The pH of the measurement buffer (10 mM NaHCO_3_, 1.5 mM KCl, and 1.0 mM NaCl) was adjusted by adding 1 M HCl or 1 M NaOH. The inhibitor experiment was performed by a previously described procedure (Horiguchi et al., 2019). For inhibitor experiments, stock solutions (10 mM) of acetazolamide (AZ) and ethoxyzolamide (EZ) were prepared by dissolution in 50 mM NaOH. AZ and EZ inhibit the external CA and internal CA, respectively. Tris(hydroxymethyl)aminomethane (TRIS), an inhibitor of HCO_3_^–^/H^+^ symport, and 4,4′-diisothiocyanatostilbene-2,2′-disulfonate (DIDS), an anion exchange protein inhibitor, were prepared daily. The photosynthetic oxygen evolution rate was measured in the HCO_3_^–^ condition (pH 8.3) in the presence of 0.1 mM AZ, 0.1 mM EZ, 50 mM TRIS, or 0.3 mM DIDS.

### Measurement of Phosphoenol Pyruvate Carboxylase, Rubisco, and Carbonic Anhydrase Activities

Terrestrial and submerged leaves were harvested 2 h after the onset of the light period and were stored at −80°C until enzymatic activities were measured. The frozen samples were homogenized in an ice-cold extraction buffer [50 mM Tris (hydroxymethyl) aminomethane (Tris), 15 mM MgCl_2_, 5 mM dithiothreitol (DTT), 0.1 mM ethylenediaminetetraacetic acid (EDTA), 10% (w/w) polyvinylpolypyrrolidone, 5% (w/v) polyvinylpyrrolidone, and 10%(v/v) glycerol (pH 8.0)] with quartz sand using a cold mortar and pestle. Part of the homogenate was used to measure the chlorophyll content; the remainder was centrifuged at 12,000 × *g*, 15 min at 4°C. The supernatant liquid (the crude extract) was kept on ice during the measurements.

Phosphoenol pyruvate carboxylase activity was determined as described previously (Zhang et al., 2014). Briefly, PEPC activity in the crude extract was measured in 1 mL of reaction mixture [50 mM Tris, 15 mM MgCl2, 0.1 mM EDTA, 20 mM bicarbonate, 0.2 mM NADH, 1 mM phosphoenol pyruvate (PEP), and 5 U malate dehydrogenase (pH 8.0)]. The reaction was started by adding PEP after preincubating the reaction mixture and sample for 5 min at 25°C. The absorbance at 340 nm was recorded for 5 min, and the activity was calculated from the change in absorbance as a function of time.

Rubisco activity was determined by modifying a previously described procedure (Suzuki et al., 2007). The Rubisco activity in crude extracts was activated by incubation with 20 mM NaHCO_3_ at 25°C for 5 min. The activated sample was injected into a reaction mixture [50 mM Tris, 15 mM MgCl_2_, 0.1 mM EDTA, 10 mM bicarbonate, 0.2 mM NADH, 0.6 mM ribulose 1,5-bisphosphate, 5.0 mM phosphocreatine, 20 U of glyceraldehyde-3-phosphate dehydrogenase, 10 U of phosphoglyceric acid kinase, and 1 U of creatine phosphokinase (pH 8.0)] that was bubbled with N_2_ gas. The activity was calculated from the decreasing absorbance at 340 nm as a function of time.

The CO_2_ hydration and HCO_3_^–^ dehydration activities of CA were determined by monitoring pH changes after the addition of substrate (Wilbur and Anderson, 1948; Kikutani et al., 2016). The CO_2_ hydration reaction was initiated by the addition of ice-cold CO_2_ saturated water to 20 mM Tris–HCl buffer (pH 8.4) containing the crude extract. The time required for the pH to drop from 8.3 to 8.0 was measured. The HCO_3_^–^ dehydration reaction was initiated by the addition of ice-cold 50 mM NaHCO_3_ to 50 mM MES buffer (pH 5.5) containing the crude extract. The time required for the pH to increase from 5.7 to 6.0 was measured. The CA activity was calculated as the Wilbur and Andersson unit (WAU): WAU = T0/T − 1, where T0 and T are the times required for the pH change in the absence and the presence of the sample, respectively.

Chlorophyll in leaf homogenates was extracted in the dark for 15 min with 80% (v/v) acetone on ice. The extract was centrifuged at 20,400 × *g* for 5 min at 25°C. The absorbance of the supernatant liquid was measured at 750, 663.6, and 646.6 nm to calculate the chlorophyll concentration (Porra et al., 1989).

### Leaf Malate Content

Malate was extracted as previously described (Yang and Liu, 2015) and quantified by an enzymatic method. Leaves were sampled at dusk (the end of the light period) or dawn (the end of the dark period). Samples were ground in ice-cold perchloric acid using an ice-cold mortar and pestle. The homogenate was kept on ice for 20 min and centrifuged at 12,000 × *g* for 30 min at 4°C. The supernatant liquid was collected, and the pellet was extracted again in ice-cold 5% (v/v) perchloric acid. The supernatant liquids from both extractions were combined, and the pH was adjusted to pH 6.5 ± 0.2 using K_2_CO_3_. After incubation on ice for 10 min, the samples were centrifuged at 12,000 × *g* for 10 min at 4°C. The supernatant liquid was filtered (0.45 μm pore size), the volume was measured, and the samples were stored at −25°C before analysis. Malate in the leaf extracts was detected by an enzymatic method using an F kit (#139068, Roche Diagnostics K.K., Tokyo, Japan).

### Anatomical and Morphological Analyses

Leaf segments (3 mm × 3 mm) added to 2% (v/v) glutaraldehyde and 2% (w/v) paraformaldehyde in 0.1 M phosphate buffer (pH 7.4) were placed in a desiccator connected to an aspirator and vacuum infiltrated to −0.09 Pa for 5 min. Infiltration was repeated until the segments were fully submerged, followed by fixation at 4°C for 3 h. The leaf segments were rinsed in 0.1 M phosphate buffer and dehydrated by an ethanol series (50, 70, 80, 90, 95, and 99.5%) followed last with acetone. The samples were infiltrated with the graded infiltration resin series, Spurr resin (Spurr Low Viscosity Embedding kit, Polysciences, Inc., PA, United States): acetone = 1:2, 1:1, 2:1 for 1.5 h, and Spurr’s resin overnight. The leaf segments were embedded in Spurr’s resin at 70°C overnight. Samples (1 μm) for light microscopy were sectioned using an ultramicrotome (Leica EM UC7, Leica, Wetzlar, Germany) at room temperature. Leaf cross sections were stained with 0.1% (w/v) toluidine blue in 0.1 M phosphate buffer (pH 7.0) and were observed using a light microscope (BX41, Olympus, Tokyo, Japan). Quantitative characteristics of leaf structure were measured using Image J ver. 1.45 s (National Institutes of Health, Bethesda, MD, United States).

Leaf morphological characteristics, including leaf area, leaf length, leaf width, and stomatal density were measured. The area, length, and width of the leaves were measured from leaf images scanned using Image J. The leaf thickness was measured from leaf cross section using Image J. Stomatal density was determined using the same microscope.

### Proteome Analysis

Proteomic analysis was performed as described previously (Perera et al., 2019). Briefly, three terrestrial (88 mg) and three submerged leaves (68 mg) were harvested. The leaves were immediately frozen in liquid N_2_ and stored at −80°C. Soluble and membrane proteins were extracted from the frozen leaves (Perera et al., 2019). Samples were analyzed using Nano liquid chromatography-mass spectrometry (LC–MS/MS) with an UltiMate 3,000 (Dionex, Tokyo, Japan) and Q-Exactive Plus (Thermo Fisher Scientific, Tokyo, Japan). LC-MS/MS data were analyzed using the search engine Mascot Server v2.5.1. Spectral data were identified against NCBInr. Statistical analysis of protein spectral counts was conducted using the proteome software Scaffold v4.8.7 (Proteosome Software, United States).

### Statistical Analyses

The data were expressed as the mean ± standard deviation (SD). Student’s *t* test was performed to detect differences between the two groups using Microsoft Excel 2013 (Microsoft, WA, United States). One-way analysis of variance (ANOVA) and Tukey’s honestly significant difference (HSD) test were calculated using JMP statistical software v9.0.2 (SAS Institute, NC, United States) and used for multiple comparisons. The initial slopes of the DIC or light response curves were generated by the Levenberg–Marquardt method and compared using the Prism software v6.07 (GraphPad Software, CA, United States). We evaluated the significance of the effect of leaf, measurement DIC concentration, or light interactions on photosynthetic rate by using two-way ANOVA; multiple comparisons were performed by the Holm–Sidak test in the Prism software v6.07. Data were determined to be statistically different when *p* < 0.05.

## Results

### Morphological Leaf Traits and Underwater Photosynthesis

**FIGURE 1:**
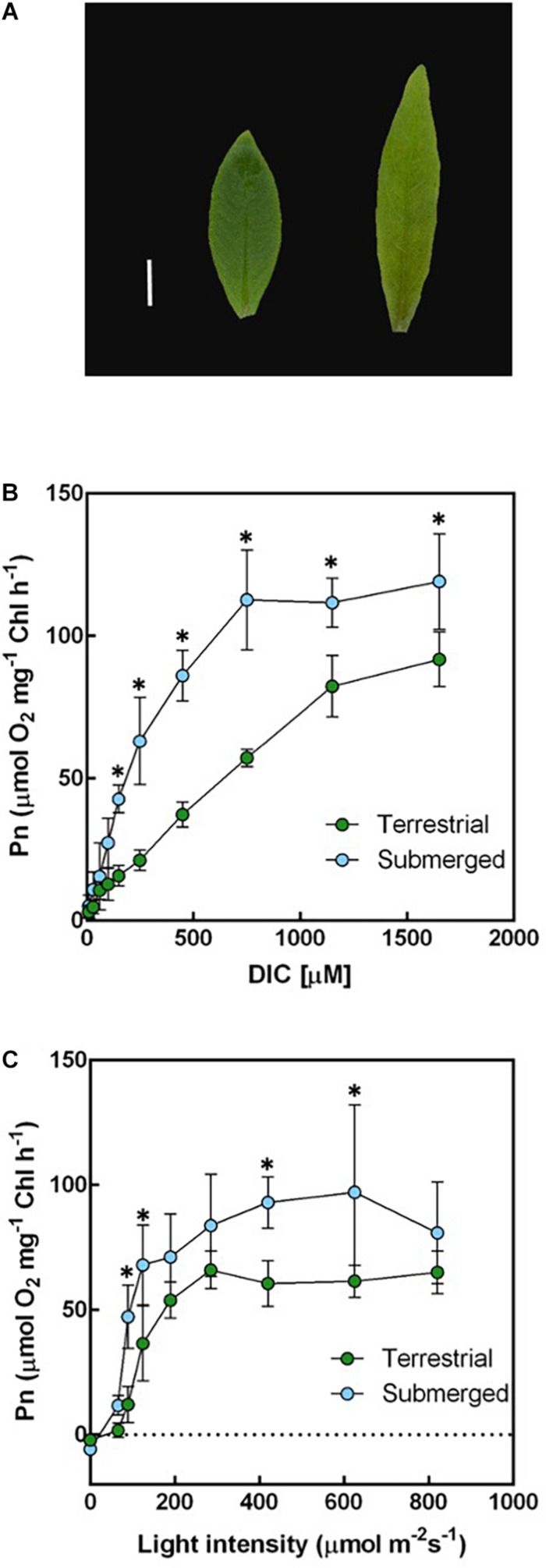
Leaf morphology, dissolved inorganic carbon (DIC), and light-response curves of underwater photosynthesis in terrestrial and submerged leaves of *Hygrophila polysperma*. **(A)** Heterophyllous leaves of *H. polysperma* that developed under terrestrial (left) or submerged environments (right). The scale bar is 10 mm. **(B)** The DIC response curve for underwater photosynthesis per chlorophyll content in the terrestrial and submerged leaves of *H. polysperma* (*n* = 3). DIC concentration increased from 10 to 1,650 μM by injected NaHCO_3_. Measurement light intensity was 285 μmol m^–2^ s^–1^, and the pH was 6.3. **(C)** Light-response curve of underwater photosynthesis per chlorophyll content in the terrestrial and submerged leaves of *H. polysperma* (*n* = 3). Photosynthetic photon flux density increased from 0 to 820 μmol photons m^–2^ s^–1^ in the presence of 10 mM NaHCO_3_ at pH 6.3. The results shown in panels **(B)** and **(C)** are expressed as means ± SD. Significance was analyzed by two-way ANOVA with Hlom–Sidak test (^∗^*p* < 0.05).

The morphology of *H. polysperma* leaves that developed under terrestrial or submerged environments was strikingly different (Figure 1A). Submerged leaves were significantly narrower and thinner compared with terrestrial leaves (Table 1). *H. polysperma* has stomata on both the abaxial and adaxial leaf surfaces. The abaxial stomatal density of submerged leaves was significantly lower than that of terrestrial leaves; however, adaxial stomatal density was not significantly different between terrestrial leaves and submerged leaves (Table 1). Submerged leaves had a significantly lower chlorophyll a and b content compared with terrestrial leaves, but the ratios of chlorophyll a to chlorophyll b (a/b) were similar (Table 1).

**TABLE 1:** Leaf morphological characteristics in terrestrial and submerged leaves of *Hygrophila polysperma*.

	**Terrestrial**	**Submerged**	**Significance**
Leaf length (mm)	28.8 ± 1.5	37.1 ± 3.7	**
Leaf width (mm)	11.8 ± 0.9	9.7 ± 0.6	**
Length-width ratio	2.4 ± 0.2	3.8 ± 0.4	**
Leaf area (mm^2^)	230.0 ± 10.3	271.8 ± 23.7	*
Leaf thickness (μm)	149.7 ± 21.6	89.3 ± 10.2	*
Abaxial stomatal density (no. mm^–2^)	92 ± 5	26 ± 3	**
Adaxial stomatal density (no. mm^–2^)	32 ± 4	24 ± 3	n.s.
Chlorophyll a (mg m^–2^)	184.9 ± 10.3	68.0 ± 10.1	**
Chlorophyll b (mg m^–2^)	66.2 ± 2.5	24.3 ± 2.4	**
Chl a/b	2.8 ± 0.05	2.8 ± 0.1	n.s.

All values are means ± SD (*n* = 3–4). Significance was analyzed with Student’s *t*-test (n.s., not significant, *p < 0.05, **p < 0.01).

We measured the net photosynthetic O_2_ evolution rate (Pn) relative to the chlorophyll content and leaf area under submerged conditions when DIC or light intensity was varied. Submerged leaves generally had a higher underwater Pn at pH 6.3 in response to an increased DIC content than terrestrial leaves at DIC concentrations above150 μM NaHCO_3_ (Figure 1B). Furthermore, the initial slopes of the DIC response curves from 10 to 150 μM NaHCO_3_ were 0.09 ± 0.02 and 0.26 ± 0.04 μmol O_2_ mg^–1^ h^–1^ μM^–1^ for terrestrial and submerged leaves, respectively (mean ± SD, *p* < 0.001). Submerged leaves had a higher underwater Pn in response to increased light intensity compared with terrestrial leaves (Figure 1C). The initial slopes of the light response curves from 0 to 125 μmol photons m^–2^ s^–1^ were 0.29 ± 0.06 and 0.60 ± 0.07 μmol O_2_ mg^–1^ h^–1^ PPFD^–1^ for terrestrial and submerged leaves, respectively (mean ± SD, *p* = 0.003). In contrast, underwater Pn per unit leaf area responded to DIC and light differently than underwater Pn expressed relative to the chlorophyll content. Terrestrial leaves had a larger underwater Pn on a per leaf area basis compared with submerged leaves at high DIC levels and high light intensities (Supplementary Figure 1). There were no significant differences in the initial slopes of underwater Pn in response to DIC content or light intensity between terrestrial and submerged leaves.

### Estimation of HCO_3_^–^ Use

**FIGURE 2:**
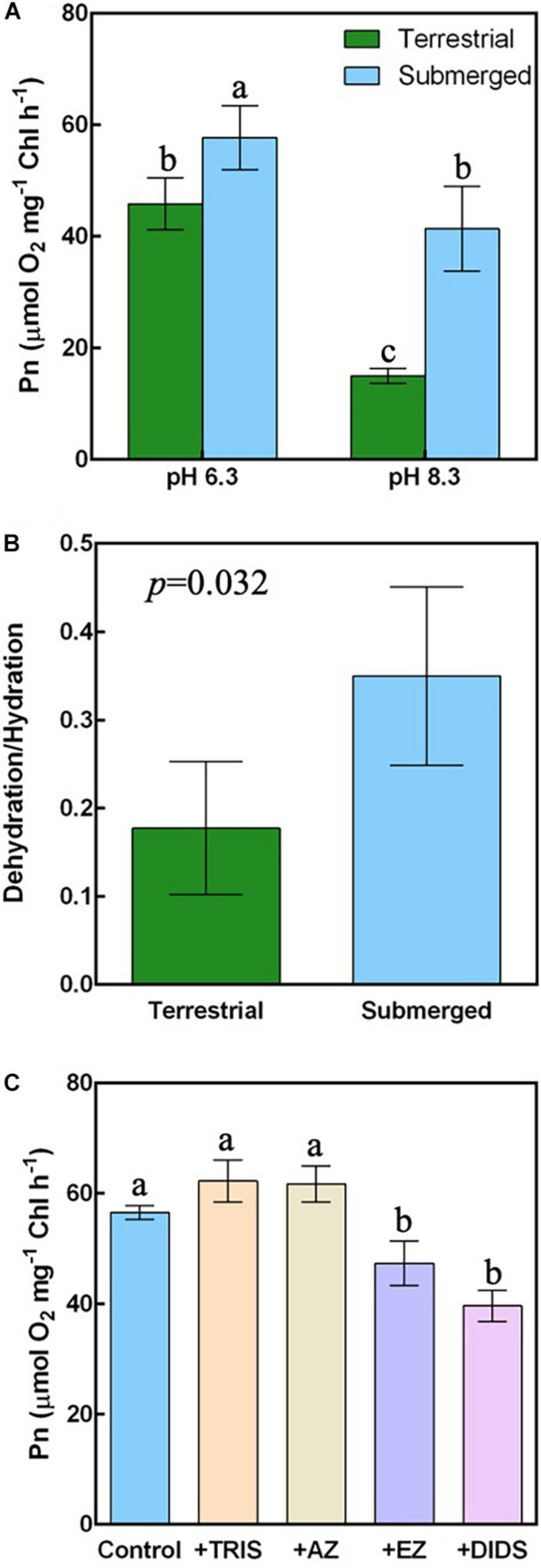
Photosynthetic HCO_3_^–^ utilization in the terrestrial and submerged leaves of *H. polysperma*. **(A)** Underwater photosynthetic rate (Pn) under different DIC conditions (*n* = 3). The DIC constituents (CO_2_ and HCO_3_^–^) in the medium at pH 6.3 were present at a ratio of 1:1 and HCO_3_^–^ only at pH 8.3, respectively. The medium contained 10 mM NaHCO_3_, 1.5 mM KCl, and 1.0 mM NaCl. **(B)** The ratio of HCO_3_^–^ dehydration to CO_2_ hydration reactions of CA (*n* = 4). **(C)** Influence of HCO_3_^–^ inhibitors on underwater Pn of the submerged leaves (*n* = 3). Underwater Pn was measured under HCO_3_^–^ conditions (pH 8.3) in the presence of 0.1 mM AZ, 0.1 mM EZ, 50 mM TRIS, or 0.3 mM DIDS. **(A,C)** Data were analyzed with Tukey’s HSD test. Different letters indicate statistical differences between the treatments (*p* < 0.05). **(B)** Data were analyzed with Student’s *t* test.

To investigate the HCO_3_^–^ affinity of *H. polysperma*, we measured the underwater Pn at pH 6.3 at which the concentrations of CO_2_ and HCO_3_^–^ are equivalent and at pH 8.3 at which the concentration of HCO_3_^–^ is dominant. The underwater Pn relative to the Chl content was highest in the submerged leaves at pH 6.3 and lowest in the terrestrial leaves at pH 8.3 (Figure 2A). The submerged leaves had a significantly higher underwater Pn compared with the terrestrial leaves in both DIC conditions. The Pn at pH 6.3 was significantly higher than at pH 8.3 in both leaf types. The HCO_3_^–^ affinity values calculated as the ratio of the Pn at pH 8.3 to the Pn at pH 6.3 were 0.3 ± 0.06 and 0.72 ± 0.11 for terrestrial and submerged leaves, respectively (mean ± SD, *p* = 0.001).

We also determined the CO_2_ hydration and HCO_3_^–^ dehydration reactions from CA activity in terrestrial and submerged leaves. The CO_2_ hydration reaction in terrestrial leaves was significantly higher than the CO_2_ hydration reaction in submerged leaves or the HCO_3_^–^ dehydration reaction in submerged and terrestrial leaves (Supplementary Figure 2). There was no difference between the CA activities other than the CO_2_ hydration reaction in terrestrial leaves. The HCO_3_^–^ dehydration/CO_2_ hydration ratio in the submerged leaves was significantly higher compared with that in the terrestrial leaves (Figure 2B).

Because submerged leaves had an increased HCO_3_^–^ affinity, we examined the mechanism of HCO_3_^–^ use by an inhibitor experiment. EZ, an inhibitor of internal CA, and DIDS, an inhibitor of the SLC family of anion exchangers, significantly inhibited the photosynthesis of submerged leaves (Figure 2C). The underwater Pns of submerged leaves was not sensitive to AZ and or TRIS, both of which inhibit external CA and HCO_3_^–^/H^+^ symport, respectively.

### Proteome Analysis

A total of 3,239 proteins were identified by proteomic analysis of which 186 proteins were differentially expressed between terrestrial and submerged leaves. One hundred proteins were significantly downregulated in submerged leaves compared with the terrestrial leaves, and 86 proteins were significantly upregulated (Supplementary Tables 1, 2). In this study, a protein identified as a HCO_3_^–^ transporter in algae was not detected in *H. polysperma*. Among seven CA proteins detected in *H. polysperma* (Table 2), the amount of chloroplast β-CA was significantly lower in the submerged leaves compared with the terrestrial leaves.

**TABLE 2:** Predicted CAs in *H. polysperma* from proteome analysis.

**Protein name**	**Accession number**	**Fold change (S/T)**	***p***
PREDICTED: LOW QUALITY PROTEIN: carbonic anhydrase, chloroplastic-like (*Malus domestica*)	XP_008348025.1	1.7	0.36
PREDICTED: carbonic anhydrase, chloroplastic-like isoform X2 (*Sesamum indicum*)	XP_011098474.1	1	0.50
PREDICTED: gamma carbonic anhydrase 2, mitochondrial (*Erythranthe guttata*)	XP_012846117.1	1.2	0.62
chloroplast carbonic anhydrase (*Pachysandra terminalis*)	ABI14813.1	INF	0.050
PREDICTED: gamma carbonic anhydrase 1, mitochondrial-like (*Phoenix dactylifera*)	XP_008810436.1 (+1)	0.7	0.41
PREDICTED: gamma carbonic anhydrase 3, mitochondrial (*Tarenaya hassleriana*)	XP_010550419.1	1	0.61
Chloroplast beta-carbonic anhydrase (*Leucaena leucocephala*)	AGS78351.1	0	0.041

*Fold change was estimated from the spectrum count. INF means infinity, protein did not detect in terrestrial leaves. The *p* values were analyzed with Fisher’s exact test.*

**FIGURE 3:**
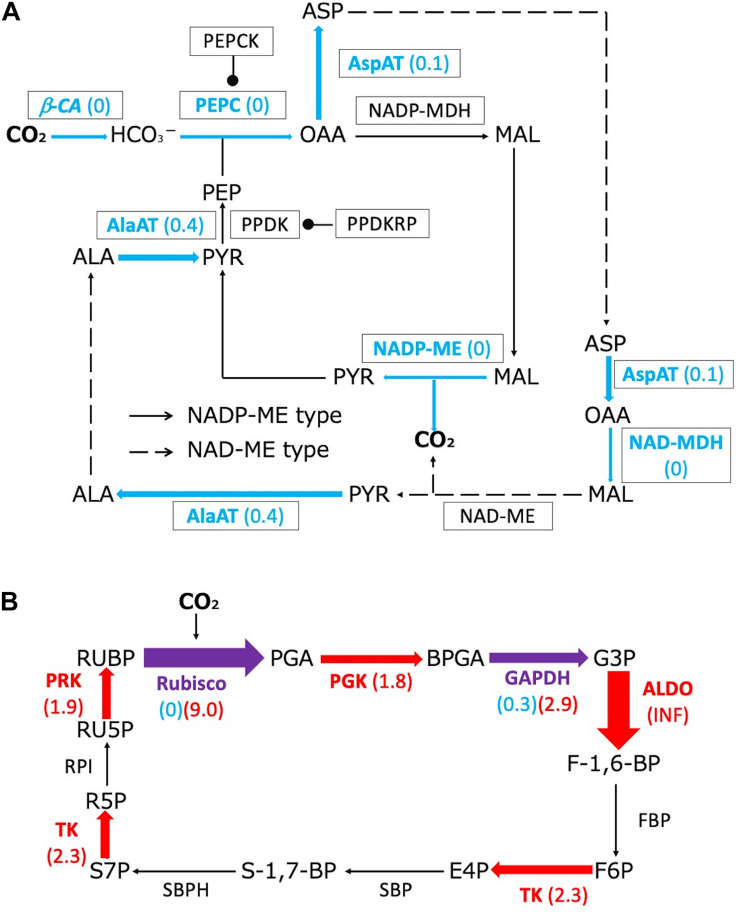
Changes in proteome profiles of C4 metabolism and Calvin cycle proteins between terrestrial and submerged leaves of *H. polysperma*. Red and blue letters and arrows indicate significantly up- and downregulated proteins in the submerged leaves compared with those produced in terrestrial leaves, respectively (Fisher’s extract test, *p* < 0.05). Purple arrows indicate proteins that were both up- and downregulated. Fold changes in protein content of the submerged leaves compared with the terrestrial leaves are shown in parentheses. **(A)** Changes in the proteome profile of C4 metabolism. Arrows and broken arrows indicate NADP ME-type and NAD ME-type metabolisms, respectively. **(B)** The changes in the proteome profile of the Calvin cycle. Metabolites: ALA, alanine; ASP, aspartate; BPGA, 1,3-bisphosphoglycerate; E4P, erythrose-4-phosphate; F-1,6-BP, fructose 1,6-bisphosphate; F6P, fructose 6-phosphate; G3P, glyceraldehyde 3-phosphate; MAL, malate; OAA, oxaloacetate; PEP, phosphoenolpyruvate; PGA, 3-phosphoglycerate; PYR, pyruvate; R5P, ribose 5-phosphate; RU5P, ribulose 5-phosphate; RUBP, ribulose 1,5-bisphosphate; S-1,7-BP, sedoheptulose-1,7-bisphosphate; S7P, sedoheptulose 7-phosphate. Enzymes: AlaAT, ALA aminotransferase; ALDO, fructose bisphosphate aldolase; AspAT, ASP aminotransferase; β-CA, beta-carbonic anhydrase; FBP, fructose-1,6-bisphosphatase; GAPDH, glyceraldehyde-3-phosphate dehydrogenase; NAD-MDH, NAD-malate dehydrogenase; NAD-ME, NAD-malic enzyme; NADP-MDH, NADP-malate dehydrogenase; NADP-malic enzyme; PEPC, PEP carboxylase; PEPCK, PEP carboxykinase; PGK, phosphoglycerate kinase; PPDK, PYR orthophosphate dikinase; PPDKRP, PPDK regulatory protein; PRK, phosphoribulokinase; RPI, ribose 5-phosphate isomerase; Rubisco, ribulose-1,5-bisphosphate carboxylase/oxygenase; SPB, sedoheptulose-bisphosphatase; SBPH, sedoheptulose 1,7-bisphosphatase; TK, transketolase.

The downregulated proteins included proteins associated with biochemical CCMs: β-CA, PEPC, aspartate aminotransferase (AspAT), NAD-malate dehydrogenase (NAD^+^-MDH), NADP-malic enzyme (NADP-ME), and alanine aminotransferase (AlaAT) (Figure 3A). Additionally, proteins related to the photosynthetic electron transport system were present in lower amounts in submerged leaves compared with terrestrial leaves (Supplementary Table 1). In contrast, the upregulated proteins included proteins associated with the Calvin cycle: Rubisco, phosphoglycerate kinase (PGK), fructose-bisphosphate aldolase (ALDO), transketolase (TK), and phosphoribulokinase (PRK) (Figure 3B). The Rubisco large subunit and glyceraldehyde-3-phosphate dehydrogenase (GAPDH) were among the proteins that were both up- and down-regulated.

### Biochemical Activities of Carbon-Concentrating Mechanisms

**FIGURE 4:**
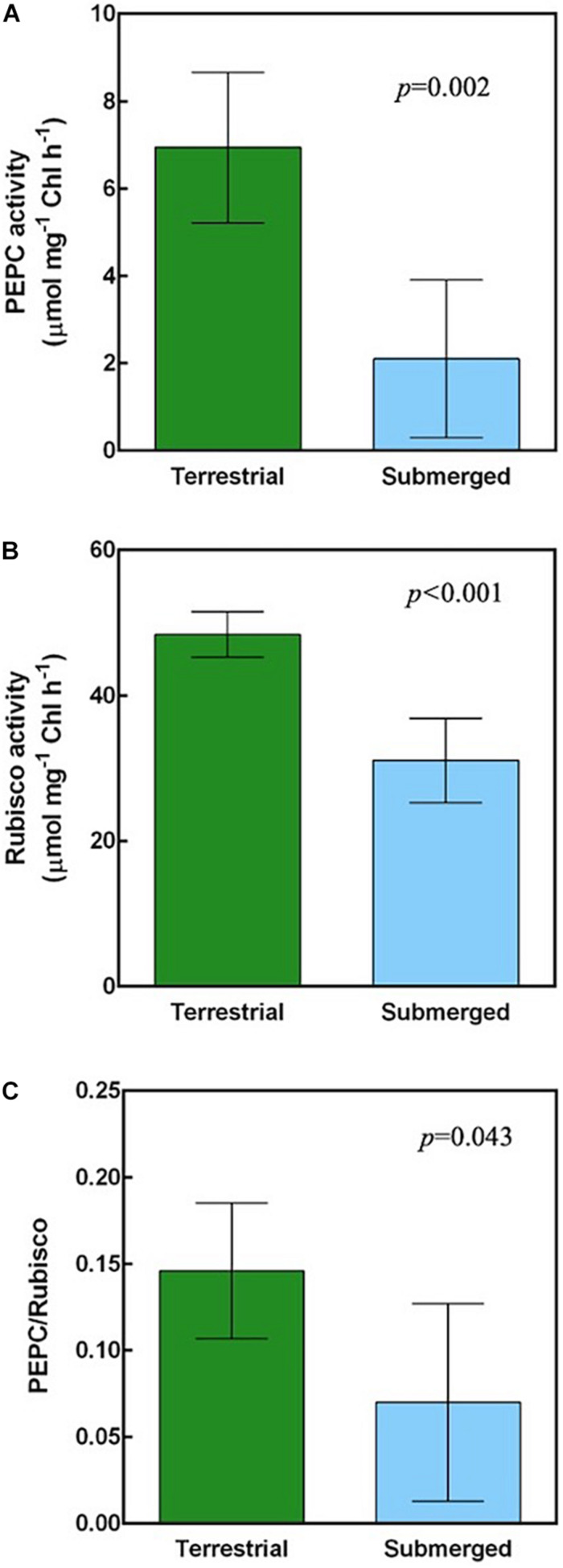
Carboxylation activities of *H. polysperma*. **(A)** PEPC activity. **(B)** Rubisco activity. **(C)** The ratio of phosphoenol pyruvate carboxylase (PEPC) to Rubisco activity. Data were analyzed with Student’s *t* test. Results are expressed as means (*n* = 5) ± SD. m, mesophyll cell; ue, upper epidermis; le, lower epidermis; bs, bundle sheath cell; a, air space. The arrowhead indicates the chloroplast.

Proteomics analysis implied that *H. polysperma* switches its photosynthetic metabolism in response to moving between terrestrial and submerged environments. We compared biochemical CCM activities between terrestrial and submerged leaves. The malate content was significantly higher at dusk than at dawn in both leaf types (Table 3). PEPC and Rubisco activities in the submerged leaves were significantly lower compared with those in the terrestrial leaves (Figures 4A,B). Moreover, submerged leaves had significantly lower PEPC/Rubisco ratios than terrestrial leaves (Figure 4C).

**TABLE 3:** Diurnal change in malate contents in terrestrial and submerged leaves of *H. polysperma*.

	**T**		**S**	
	**Dawn**	**Dusk**	**Dawn**	**Dusk**
Malate content (μmol g^–1^ f.w.)	2.3 ± 0.3^a^	4.2 ± 0.4^b^	2.4 ± 0.4^a^	3.6 ± 0.3^b^

*The leaves were harvested at dusk (the end of light period, white bar) or dawn (the end of dark period, gray bar). Data analyzed with Tukey’s HSD test. Different letters indicate statistical differences between the leaves (*p* < 0.05). Results are expressed as means ± SD (*n* = 4).*

### Leaf Anatomical Traits Observed by Light Microscopy

**FIGURE 5:**
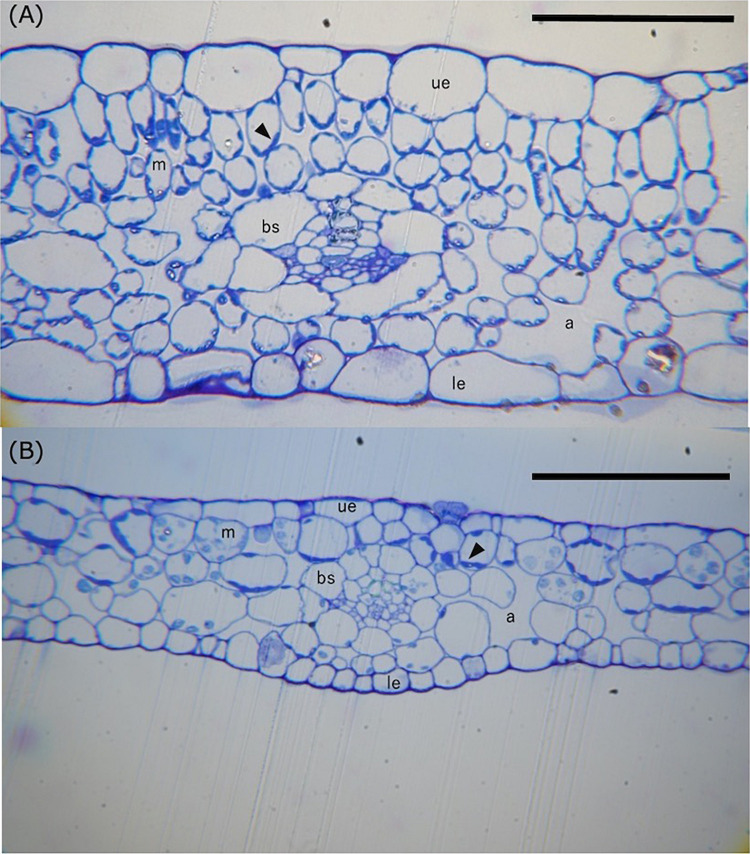
Light photomicrographs of leaf cross sections of the terrestrial **(A)** and submerged **(B)** leaves of *H. polysperma*. Scale bars = 100 μm. m, mesophyll cell; ue, upper epidermis; le, lower epidermis; bs, bundle sheath cell; a, air space. The arrowhead indicates the chloroplast.

Figure 5 compares the anatomy of leaf cross sections prepared from *H. polysperma* terrestrial and submerged leaves. Terrestrial leaves had cells that varied in both shape and size. In cross section, the upper layer of MCs in terrestrial leaves were vertically long, whereas those in the lower layer were horizontally long or spherical (Figure 5A). In contrast, submerged leaves had horizontally long or spherical cells but not vertically long cells (Figure 5B). We observed that both upper and lower MCs in terrestrial leaves have a chloroplast; however, some lower MCs in submerged leaves had no chloroplast (Figures 5A,B). Chloroplasts were absent in the epidermal cells of both terrestrial and submerged leaves. Kranz anatomy was evident in cross sections of terrestrial leaves but not in submerged leaves (Figures 5A,B); however, chloroplasts in BSCs were not located near the vascular bundles as is typical for the Kranz anatomy in some C4 plants.

**FIGURE 6:**
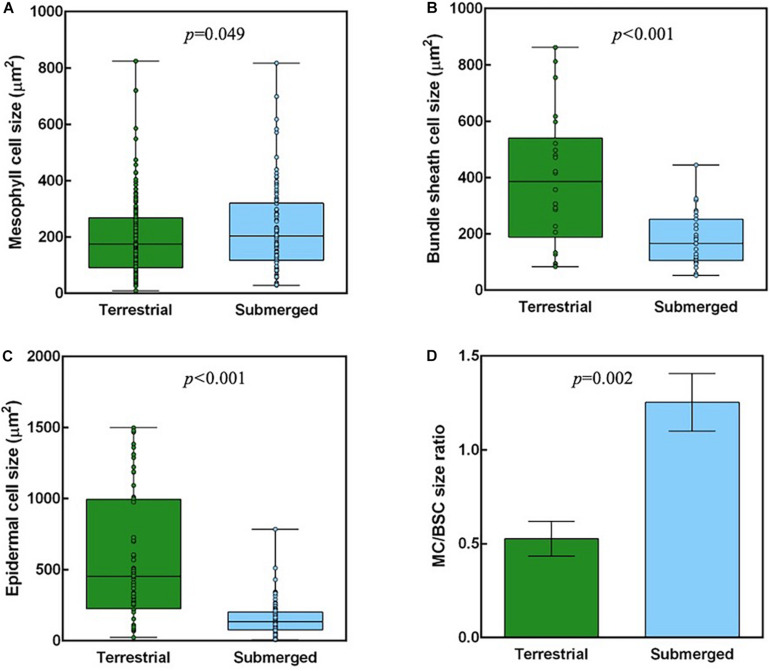
Anatomical measurements of *H. polysperma* leaves that developed under terrestrial and submerged conditions. Cell sizes of mesophyll cells **(A)**; bundle sheath cells **(B)**; epidermal cells **(C)** and the size ratio of mesophyll cells (MCs) to Rubisco in bundle sheath cells (BSCs) **(D)**. **(A–C)** Cell sizes were measured for all cells in the profile area (W370 μm) from three independent biological replicates. Boxes indicate the 25th and 75th percentiles, with medians indicated by the horizontal line in the box. The open circles in the boxes show the raw data. **(D)** The ratio of MC to BSC size was calculated from averages of MC and BSC sizes in each sample. The data were analyzed with Student’s *t* test, and the results are expressed as means ± SD.

The analysis of cross sections from terrestrial and submerged leaves identified significant differences in leaf structure (Figure 6). The MC size was significantly higher in submerged leaves than in terrestrial leaves (Figure 6A). The BSC and epidermal cell sizes were significantly smaller in the submerged leaves compared with the terrestrial leaves (Figures 6B,C). Therefore, the submerged leaves had a significantly higher MC/BSC size ratio compared with terrestrial leaves (Figure 6D). On the other hand, there were no differences in the percent of tissue coverage of a cross section between terrestrial and submerged leaves (Supplementary Table 3).

## Discussion

### HCO_3_^–^ Uptake and Conversion to CO_2_

HCO_3_^–^ use mechanisms are distinguished by inhibitor sensitivity in underwater photosynthesis. In a previous study, four inhibitors of HCO_3_^–^ utilization (AZ, TRIS, EZ, and DIDS) were used to distinguish HCO_3_^–^ use mechanisms in aquatic phototrophs (Beer et al., 2002; Hellblom and Axelsson, 2003; Fernández et al., 2014; Rubio et al., 2017; Tsuji et al., 2017). In marine diatoms, DIDS, an anion exchanger inhibitor, suppressed high-DIC-affinity photosynthesis in the pennate diatom *P. tricornutum* and the centric diatom *Chaetoceros muelleri*, but AZ, an external CA inhibitor, did not affect high-DIC-affinity photosynthesis (Tsuji et al., 2017). Tsuji et al. (2017) also reported that AZ reduced HCO_3_^–^ affinity in the pennate *Cylindrotheca fusiformis* and the centric *Thalassiosira pseudonana*, but DIDS did not affect HCO_3_^–^ affinity. The DIDS-sensitive group converted HCO_3_^–^ to CO_2_ after transport of HCO_3_^–^ into cells; the DIDS-insensitive group facilitated CO_2_ diffusion by external CA. Submerged leaves of *H. polysperma* had an enhanced affinity for HCO_3_^–^ compared with terrestrial leaves (Figure 2A). The ratio of HCO_3_^–^ dehydration to CO_2_ hydration by CA was higher in the submerged leaves than in the terrestrial leaves (Figure 2B). Furthermore, EZ, internal CA inhibitor, and DIDS inhibited the underwater Pn of submerged leaves, AZ and TRIS; HCO_3_^–^/H^+^ symporter inhibitor did not (Figure 2C). These results suggest that *H. polysperma* transports HCO_3_^–^ into leaves by a DIDS-sensitive transporter and converts HCO_3_^–^ to CO_2_ by an intercellular CA. *H. polysperma* leaves grown under submerged conditions achieved a higher photosynthetic rate by a biophysical CCM in low DIC levels (Figure 1B); therefore, submerged plants accumulated more proteins associated with the Calvin cycle compared with plants grown under terrestrial conditions (Figure 3B). HCO_3_^–^ use mechanism in *H. polysperma* is an effective way to acclimate to submerged environment.

4,4′-Diisothiocyanatostilbene-2,2′-disulfonate inhibits HCO_3_^–^ uptake and/or underwater photosynthesis in plants ranging from algae to higher plants (Nakajima et al., 2013; Fernández et al., 2014; Huang et al., 2020). Huang et al. (2020) first reported the presence of direct HCO_3_^–^ uptake *via* a DIDS-sensitive SLC4 transporter in the higher plant *O. alismoides*. Here, we report that the underwater Pn of *H. polysperma*-submerged leaves was also inhibited by DIDS; this is the first report of DIDS inhibiting the photosynthetic gas exchange of a higher plant. DIDS inhibits not only the SLC4 family of transporters but also members of the SLC26 gene family in mammals (Soleimani et al., 2001). HCO_3_^–^ transporters associated with biophysical CCMs have been identified in both the SLC4 and SLC26 gene families; SLC4 is present in a marine diatom (Nakajima et al., 2013) and an aquatic angiosperm (Huang et al., 2020), and BicA, a member of the SLC26/SulP transporter family, is present in cyanobacteria (Price and Howitt, 2011). Nevertheless, neither SLC4 nor SLC26 anion exchangers annotated as HCO_3_^–^ transporter were identified in our proteomics analysis without regard to the expression levels. These results suggest that *H. polysperma* may move HCO_3_^–^ into leaves *via* a novel and as yet unidentified DIDS-sensitive HCO_3_^–^ transporter.

In algae, inorganic carbon uptake within the cells consumes light energy (Tchernov et al., 2003; Burnap et al., 2015; Wang et al., 2015). To photosynthesize in MCs, *H. polysperma* transports HCO_3_^–^ into leaves *via* the epidermal cells. Because *H. polysperma* does not have chloroplasts in the epidermal cells (Figure 5), energy for HCO_3_^–^ uptake may have to be supplied by MCs. Submerged leaves had higher initial slopes of their photosynthetic light response curves on a chlorophyll basis, indicating more efficient light use (Figure 1C). The chlorophyll content per leaf area in the submerged leaves was significantly lower than that in the terrestrial leaves, but the light use efficiency on the basis of leaf area was constant (Table 1 and Supplementary Figure 1B). These results imply that the light use efficiency of submerged leaves was high to provide sufficient light energy for HCO_3_^–^ uptake.

In algae, CA is a key component of the biophysical CCMs. In algae, *CA* mutants have lower photosynthetic DIC affinity and growth in low CO_2_ conditions than wild type (Karlsson et al., 1998; Kikutani et al., 2016). These CAs are localized in the thylakoid lumen. Chloroplast CAs that are localized in the thylakoid lumen are assumed to supply CO_2_ to the Calvin cycle. CA contributes not only biophysical CCMs but also biochemical CCMs. In C4 plants, CO_2_ hydration activity of CA plays an essential role in primary CO_2_ fixation by HCO_3_^–^ production. Some studies revealed the CA contributions of MC and BSC to total CA activity. *Amaranthus cruentus* leaves and C4 eudicots, were isolated two CAs (Guliev et al., 2003). One associated with the chloroplast of BSCs is responsible for 8% of the total leaf CA activity; the other, found in the MC cytoplasmic fraction, represents 62% of the total leaf CA activity (Guliev et al., 2003). In corn (*Zea mays*, C4 grass) and green foxtail millet [*Setaria viridis* (L) P. Beauv., C4 grass], CO_2_ assimilation rates in transgenic lines of loss of β-CA(s) were unchanged at ambient CO_2_ but decreased at low CO_2_ (Studer et al., 2014; Osborn et al., 2017). The β-*CA* mutant of *S. viridis* decreases growth and photosynthesis at ambient CO_2_ (Chatterjee et al., 2021). On the other hand, pineapple (*Ananas comosus*), a CAM plant, increases the expression levels of three copies of CA at night time (Ming et al., 2015). Cytosolic β-CA activity may be partial to CO_2_ hydration reaction contrary to chloroplast CA. The submerged leaves of *H. polysperma* increased the HCO_3_^–^ dehydration/CO_2_ hydration ratio of CA activity compared with that of terrestrial leaves (Figure 2B); however, the terrestrial leaves attained higher HCO_3_^–^ dehydration and CO_2_ hydration CA activities than those of the submerged leaves (Supplementary Figure 2). Seven differentially regulated proteins were identified as CAs by proteomic analysis (Table 2). The amount of chloroplast β-CA protein was significantly lower in the submerged leaves than in the terrestrial leaves, whereas the levels of other CAs did not change. In the case of other aquatic plants, *P. lucens* lack external CA (Staal et al., 1989); CA activity of *E. canadensis* is not influenced by the CO_2_ concentration (Elzenga and Prins, 1988). In *O. alismoides*, external CA activity was greater in leaves acclimated to low CO_2_ aquatic environment compared with that in leaves acclimated to high CO_2_ aquatic environment; however, the expression of the four isoforms of putative α-*CA1* was not significantly different between two CO_2_ conditions (Huang et al., 2020). Our results suggest that the reduction in CO_2_ hydration activity in the submerged leaves is caused by a decrease in the amount of β-CA, resulting in an increased HCO_3_^–^ dehydration/CO_2_ hydration ratio of CA activity in the submerged leaves.

### Structural Changes in Terrestrial and Submerged Leaves

Aquatic plants have different leaf anatomical characteristics compared with land plants: thinner leaves, cuticles, and cell walls, a few or no stomata, and epidermal cells with chloroplasts or chloroplasts facing the epidermis in MCs (Veen and Sasidharan, 2019). The thin leaves of aquatic plants improve gas exchange when leaves are submerged (Frost-Christensen et al., 2003). The submerged leaves of *H. polysperma* were thinner than the terrestrial leaves, and the stomatal density on the abaxial surface was lower than that of the terrestrial leaves (Table 1). The reduction in leaf thickness was achieved by decreasing the thickness of the MC layer (Figures 5A,B). Considering the results, *H. polysperma* appears to follow an acclimation mechanism for aquatic plants that facilitates passive CO_2_ diffusion. Furthermore, both molecular size and diffusion resistance of HCO_3_^–^ are larger than that of CO_2_, thinner leaves, especially smaller epidermal cells (Figure 6C), are effective not only in CO_2_ diffusion but also in HCO_3_^–^ uptake. The submerged leaves had large MCs compared with terrestrial leaves (Figures 5A,B, 6A). These MCs in submerged leaves are expected to equip with big vacuoles. In submerged leaves of *H. polysperma*, some lower MCs were observed the absence of chloroplasts (Figure 5B). The submerged leaves may separate function between upper and lower MCs as in the cases of *E. canadensis* and *P. lucens*. For instance, upper MCs, which contain chloroplasts, perform photosynthesis using HCO_3_^–^; lower MCs, which have no or few chloroplasts, excrete or store OH^–^ to maintain pH homeostasis.

### Terrestrial *Hygrophila polysperma* Had Proto-Kranz-Type Photosynthetic Characteristics

In this study, we found that the submerged leaves of *H. polysperma* accumulated lower levels of proteins associated with biochemical CCM compared with the terrestrial leaves (Figure 3A). A result of the proteomics analysis implies that the terrestrial leaves use a biochemical CCM, and the submerged leaves do not. Biochemical CCMs include C4 photosynthesis and CAM, two pathways that require specific enzymes. Therefore, we measured the diel changes in malate content to confirm CAM activity in the leaves of *H. polysperma*. Terrestrial and submerged leaves did not accumulate malate at dawn (Table 3), suggesting that the terrestrial leaves did not perform CAM. Modified C4 photosynthetic pathways are identified as C3–C4 and C4-like photosynthesis in some plant species such as *Flaveria* (Mckown and Dengler, 2007), *Eleocharis* (Murphy et al., 2007), *Heliotropium* (Muhaidat et al., 2011), *Moricandia* (Schlüter et al., 2017), and *Chenopodium* (Yorimitsu et al., 2019). C3–C4 intermediate plants have extended BSCs and lower CO_2_ compensation point. In *Eleocharis*, C3–C4 intermediate species exhibit weak C4 cycles that means that there is a higher rate of PEPC to Rubisco than that of the C3 species and localization of enzymes between MC and BSC (Murphy et al., 2007). In *Heliotropium* and *Cheopodium* C3–C4 intermediate species, abundant mitochondria and localization of glycine decarboxylase in extended BSCs suggested that they capture, concentrate, and re-assimilate CO_2_ released by photorespiration (Muhaidat et al., 2011; Yorimitsu et al., 2019). This simple CCM is called C2 photosynthesis, glycine shuttle, and photorespiratory CO_2_ pump (Lundgren, 2020). Interestingly, some *Heliotropium* and *Chenopodium* species exhibit a proto-Kranz type of photosynthesis that is functionally C3 but involves BSCs. Proto-Kranz plants have larger BSC size than MC size like the biochemical CCM plant; however, enzyme activities related to C4 photosynthesis are the same levels as C3 plants (Muhaidat et al., 2011; Yorimitsu et al., 2019). The biochemical and anatomical characteristics of *H. polysperma* leaves had unique features that do not differentiate between photosynthetic types. The ratio of PEPC to Rubisco increases with the transition from C3 to C4, leading to enhanced C4 activity. In the terrestrial leaves of *H. polysperma*, the ratio of PEPC to Rubisco was significantly higher than that in the submerged leaves (Figure 4C). However, the ratios of PEPC to Rubisco were 0.29 and 0.14 in the terrestrial and submerged leaves, a range in values that is typical for C3 plants (Murphy et al., 2007; Zhang et al., 2014). Anatomical analysis of *H. polysperma* found vascular bundles surrounding BSCs that contained few chloroplasts (Figures 5A,B). Nevertheless, the ratio of MC/BSC in a cross sectional area was 4.5 in the terrestrial and submerged leaves (Supplementary Table 3), a value that is identical to that in *Flaveria*, a C3–C4 plant (Mckown and Dengler, 2007), *Heliotropium* (Muhaidat et al., 2011), and the Kranz-like plant *Chenopodium* (Yorimitsu et al., 2019). In *H. polysperma*, the cell size ratios of MC/BSC were significantly different between terrestrial and submerged leaves (Figure 6D) at 0.5 and 1.3, respectively. These ratios are within the range for proto-Kranz- and non-Kranz-type plants (Yorimitsu et al., 2019). Our results imply that under terrestrial conditions, *H. polysperma* conducts incomplete C4 photosynthesis, likely proto-Kranz, and alters its photosynthetic metabolism to C3 photosynthesis when leaves are underwater.

### Conclusion

In summary, we report a comprehensive photosynthetic mechanism to acclimate *H. polysperma*, a heterophyllous amphibious plant to a submerged environment that has limited CO_2_. Leaves that developed in the submerged condition adopted two DIC uptake strategies: (i) passive CO_2_ diffusion without external CA and (ii) active HCO_3_^–^ transport by a DIDS-sensitive HCO_3_^–^ transporter-like member of the SLC gene family. Furthermore, our results suggest that *H. polysperma* can alter its photosynthesis from a proto-Kranz type in terrestrial leaves to a C3 type in submerged leaves. To improve photosynthetic performance for increasing crop yields, many researchers have attempted to introduce a biophysical CCM or a C4 pathway into C3 crop plants (Price et al., 2013; Ermakova et al., 2020). Further characterization and understanding of the acclimation mechanisms to extreme stress conditions in *H. polysperma* will provide novel resources for improving stress resistance and photosynthesis in eudicot crop plants.

## Data Availability Statement

The datasets presented in this study can be found in online repositories. The names of the repository/repositories and accession number(s) can be found below: ProteomeXchange (PXD024779) and jPOST (JPST001108).

## Author Contributions

GH and NH conceived the research project and designed the study. GH, KM, and KN performed the experiments and analyzed the data. MI supported the anatomical analysis. GH and NH wrote manuscript. All authors contributed to the article and approved the submitted version.

## Conflict of Interest

The authors declare that the research was conducted in the absence of any commercial or financial relationships that could be construed as a potential conflict of interest.
