# Supplementary material for: Transition From Proto-Kranz-Type Photosynthesis to HCO3– Use Photosynthesis in the Amphibious Plant Hygrophila polysperma
Source: Front Plant Sci. 2021 Jun 16;12:675507. doi: 10.3389/fpls.2021.675507 (PMC8242947; doi:10.3389/fpls.2021.675507)
Supplement: Supplementary file 1 [file Data_Sheet_1.PDF]

Supplementary Table S1. Proteins accumulating in significantly lower amounts in submerged leaves compared to terrestrial leaves.

Protein Name	Accession Number	Fold Change (S / T)	*p*
PREDICTED: glucan endo-1,3-beta-glucosidase, acidic isoform PR-Q'-like [*Erythranthe guttata*]	XP_012845506.1	0	< 0.00010
PREDICTED: catalase isozyme 3 [*Cucumis melo*]	XP_008464839.1	0	< 0.00010
ATP synthase CF1 alpha subunit (chloroplast) *[Trithuria filamentosa*]	AHB38639.1 (+1)	0	0.00034
PREDICTED: ABC transporter B family member 4-like [*Sesamum indicum*]	XP_011079478.1	0	0.00076
hypothetical protein CARUB_v10026803mg [*Capsella rubella*]	XP_006279655.1 (+1)	0	0.00076
hypothetical protein CICLE_v10027881mg [*Citrus clementina*]	XP_006424585.1	0	0.00076
PREDICTED: polyphenol oxidase I, chloroplastic-like [*Sesamum indicum*]	XP_011098121.1	0	0.0017
beta-1,3-glucanase, basic [Coffea arabica x Coffea canephora]	AAQ90286.1 (+4)	0	0.0017
Glutathione S-transferase/chloride channel, C-terminal [*Cynara cardunculus* var. scolymus]	KVI03991.1	0	0.0017
PREDICTED: pectinesterase-like isoform X1 [Sesamum indicum]	XP_011078159.1 (+1)	0	0.0017
hypothetical protein SELMODRAFT_132915 [*Selaginella moellendorffii*]	XP_002991055.1 (+1)	0	0.0017
PREDICTED: 1,4-dihydroxy-2-naphthoyl-CoA synthase, peroxisomal [*Sesamum indicum*]	XP_011071094.1 (+1)	0	0.0017
PREDICTED: catalase [*Beta vulgaris* subsp. vulgaris]	XP_010692546.1	0	0.0038
betaine aldehyde dehydrogenase [*Ligusticum sinense*]	ADL61811.1	0	0.0038
PREDICTED: peroxidase 15-like [*Sesamum indicum*]	XP_011078985.1	0	0.0038
PREDICTED: dirigent protein 22-like [*Vitis vinifera*]	CBI29983.3 (+1)	0	0.0038

gibberellin 20-oxidase 2 [*Citrus sinensis* x *Citrus trifoliata*]	ACJ54434.1 (+2)	0	0.0038
PREDICTED: formate dehydrogenase, mitochondrial-like [*Eucalyptus grandis*]	XP_010033275.1	0	0.0083
PREDICTED: purple acid phosphatase-like [*Elaeis guineensis*]	XP_010910529.1 (+1)	0	0.0083
cell-wall invertase [*Phelipanche ramosa*]	ADF27781.1 (+2)	0	0.0083
hypothetical protein SELMODRAFT_429447 [*Selaginella moellendorffii*]	XP_002991114.1 (+1)	0	0.0083
hypothetical protein JCGZ_02770 [*Jatropha curcas*]	KDP39750.1 (+1)	0	0.019
NADP-dependent malic enzyme [*Prunus humilis*]	ALI57372.1	0	0.019
PREDICTED: lignin-forming anionic peroxidase-like [*Musa acuminata* subsp. malaccensis]	XP_009418659.1	0	0.019
chloroplast beta-carbonic anhydrase [*Leucaena leucocephala*]	AGS78351.1	0	0.041
Peptidyl-prolyl cis-trans isomerase FKBP16-4, chloroplastic -like protein [*Gossypium arboreum*]	KHG27779.1 (+6)	0	0.041
macrophage migration inhibitory factor family protein / MIF family protein [*Sonneratia alba*]	ABQ42101.1 (+46)	0	0.041
PREDICTED: peroxidase 4-like [*Nelumbo nucifera*]	XP_010264466.1	0	0.041
PREDICTED: elongation factor 2 [*Sesamum indicum*]	XP_011095694.1	0	0.041
Plant uncoupling mitochondrial protein 1 [*Theobroma cacao*]	XP_007026812.1 (+1)	0	0.041
hypothetical protein M569_05124, partial [*Genlisea aurea*]	EPS69640.1	0	0.041
PREDICTED: beta-xylosidase/alpha-L-arabinofuranosidase 2-like [*Sesamum indicum*]	XP_011092407.1	0	0.041
PREDICTED: spermidine synthase 2 [*Camelina sativa*]	XP_010427802.1	0	0.041
class IV chitinase Chia4-Pa1.3 [*Picea abies*]	AAQ17048.1 (+12)	0	0.041
ribulose 1,5-bisphosphate carboxylase [*Cannabis sativa*]	CAC04295.1	0	0.041
unknown [*Glycine max*]	ACU19296.1 (+10)	0	0.041
cytochrome b-559 alpha subunit protein, partial (chloroplast) [*Scouleria aquatica*]	AFJ73211.1	0	0.041
phosphoenolpyruvate carboxylase [*Manihot esculenta* subsp. flabellifolia]	AFN70425.1	0	0.041
predicted protein [*Hordeum vulgare* subsp. vulgare]	BAK05511.1 (+1)	0	0.041
PREDICTED: alanine aminotransferase 2, mitochondrial isoform X1or2	KJB57090.1 (+2)	0	0.041

2-oxoglutarate dehydrogenase, E1 component isoform 1 [*Theobroma cacao*]	XP_007009419.1 (+1)	0	0.041
PREDICTED: peroxidase 25 [*Nicotiana sylvestris*]	XP_009779949.1	0	0.041
PREDICTED: malate dehydrogenase, mitochondrial [*Populus euphratica*]	XP_011006727.1	0	0.041
ATP synthase CF1 beta subunit (chloroplast) [*Chrysanthemum* x *morifolium*]	YP_007353773.1 (+1)	0	0.041
cysteine protease [*Salix matsudana*]	AGN96213.1 (+2)	0	0.041
hypothetical protein LR48_Vigan01g082000 [Vigna angularis]	KOM31265.1 (+7)	0	0.041
PREDICTED: (+)-neomenthol dehydrogenase-like [*Cicer arietinum*]	XP_012574180.1	0	0.041
RecName: Full=Catalase [*Avicennia marina*]	Q9AXH0.1	0.08	< 0.00010
PREDICTED: uncharacterized protein At5g01610 [*Sesamum indicum*]	XP_011082452.1	0.1	0.0045
citrate synthase, partial [*Genlisea aurea*]	EPS67962.1	0.1	0.0045
PREDICTED: aspartate aminotransferase, mitochondrial [*Prunus mume*]	XP_008228936.1	0.1	0.0091
hypothetical protein EUGRSUZ_I024522, partial [*Eucalyptus grandis*]	KCW56781.1 (+1)	0.1	0.0091
PREDICTED: pectinesterase-like [*Nelumbo nucifera*]	XP_010261668.1	0.1	0.018
NADP-dependent malic enzyme [*Morus notabilis*]	XP_010102421.1	0.2	< 0.00010
PREDICTED: catalase isoform X1 [*Sesamum indicum*]	XP_011074090.1	0.2	< 0.00010
PREDICTED: peroxidase 25 [*Brassica oleracea* var. oleracea]	XP_013634178.1 (+2)	0.2	0.00015
beta-glucosidase [*Populus tomentosa*]	AFZ78537.1 (+1)	0.2	0.0019
PREDICTED: GDSL esterase/lipase 4-like [*Eucalyptus grandis*]	XP_010029492.1 (+1)	0.2	0.0035
Peroxidase 51 -like protein [*Gossypium arboreum*]	KHG21770.1 (+8)	0.2	0.0042
Beta-glucosidase, family GH1 [*Zostera marina*]	KMZ71222.1	0.2	0.028
Reticuline oxidase precursor, putative [*Ricinus communis*]	EEF39187.1 (+41)	0.2	0.028
PREDICTED: protein ASPARTIC PROTEASE IN GUARD CELL 2-like [*Solanum lycopersicum*]	XP_004229990.1 (+3)	0.2	0.036

PREDICTED: peroxidase 5-like [*Solanum lycopersicum*]	XP_004251979.1 (+2)	0.2	0.036
PREDICTED: NADH dehydrogenase [ubiquinone] iron-sulfur protein 1, mitochondrial [*Ricinus communis*]	XP_002531931.1	0.2	0.036
PREDICTED: peroxidase 4-like [*Erythranthe guttata*]	XP_012840620.1	0.3	< 0.00010
RecName: Full=Catalase [*Helianthus annuus*]	P45739.1 (+1)	0.3	< 0.00010
ATP synthase CF1 alpha subunit (chloroplast) [*Salix interior*]	YP_009051547.1 (+2)	0.3	0.00014
unnamed protein product [*Coffea canephora*]	CDP07707.1	0.3	0.0028
elongation factor [*Saccharum officinarum*]	ABU63160.1	0.3	0.0087
hypothetical protein LR48_Vigan05g030700 [*Vigna angularis*]	KOM42703.1	0.3	0.02
PREDICTED: ADP,ATP carrier protein 1, mitochondrial (=ADP/ATP translocase 1)[*Cucumis sativus*]	XP_004142869.2	0.3	0.036
glyceraldehyde 3-phosphate dehydrogenase [*Sedum alfredii*]	AHB86970.1	0.3	0.036
PREDICTED: peroxisomal (S)-2-hydroxy-acid oxidase GLO1 [*Vitis vinifera*].	CAN74334.1 (+2)	0.4	< 0.00010
photosystem II oxygen-evolving enhancer protein 1	CDP02430.1	0.4	0.00065
PREDICTED: peroxidase 73-like [*Nelumbo nucifera*]	XP_010272213.1	0.4	0.007
PREDICTED: pyruvate dehydrogenase E1 component subunit beta-1, mitochondrial [*Sesamum indicum*]	XP_011073116.1	0.4	0.015
PREDICTED: cytochrome c1-2, heme protein, mitochondrial-like [*Solanum lycopersicum*]	XP_004252324.1 (+2)	0.4	0.022
PREDICTED: lysosomal beta glucosidase-like [*Sesamum indicum*]	XP_011077752.1	0.4	0.026
PREDICTED: cytochrome b6-f complex iron-sulfur subunit 1, chloroplastic [*Vitis vinifera*]	XP_002284361.1	0.4	0.03
PREDICTED: alanine aminotransferase 2 [*Sesamum indicum*]	XP_011072689.1	0.4	0.043

Supplementary Table S2. Proteins accumulating significantly higher amounts in submerged leaves compared to terrestrial leaves. INF means infinity, protein did not detect in terrestrial leaves.

Protein Name	Accession Number	Fold Change (S / T)	*p*
PREDICTED: glucose-1-phosphate adenylyltransferase small subunit, chloroplastic/amyloplastic-like [*Sesamum indicum*]	XP_011086297.1 (+1)	INF	0.00013
PREDICTED: probable aldo-keto reductase 2 [*Ricinus communis*]	CDP10300.1	INF	0.00023
stem-loop-binding protein of 41 kDa protein A [*Medicago truncatula*]	XP_003590649.1	INF	0.0014
PREDICTED: geraniol 8-hydroxylase-like [*Sesamum indicum*]	XP_011099226.1	INF	0.0025
PREDICTED: fasciclin-like arabinogalactan protein 17 [*Sesamum indicum*]	XP_011094205.1	INF	0.0025
putative glucose-6-phosphate isomerase, partial [*Taraxacum brevicorniculatum*]	AIU34465.1	INF	0.0025
chalcone isomerase, partial [*Scutellaria lateriflora*]	AHE41428.1	INF	0.0025
granule-bound starch synthase, partial [*Solanum quadriloculatum*]	ADE40957.1	INF	0.0046
Rab2 [*Hevea brasiliensis*]	AEA92304.1 (+55)	INF	0.0046
PREDICTED: LOW QUALITY PROTEIN: plasma membrane ATPase 3 [*Sesamum indicum*]	XP_011071717.1	INF	0.0046
PREDICTED: alpha-glucan phosphorylase, H isozyme [*Sesamum indicum*]	XP_011069887.1	INF	0.0046
ribosomal protein S7 (chloroplast) [*Triantha glutinosa*]	AAN31967.1 (+122)	INF	0.0083
ribulose-1,5-bisphosphate carboxylase/oxygenase large subunit [*Pimelea longiflora* subsp. eyrei]	ADI16181.1	INF	0.0083
biotin carboxylase [*Camellia chekiangoleosa*]	AGH32911.1	INF	0.0083
ribulose bisphosphate carboxylase small chain [*Medicago truncatula*]	XP_013447458.1	INF	0.0083

PREDICTED: oligopeptide transporter 3 [*Nicotiana tomentosiformis*]	XP_009590288.1 (+1)	INF	0.0083
hypothetical protein PHAVU_007G224500g [*Phaseolus vulgaris*]	XP_007145267.1	INF	0.0083
fructose-bisphosphate aldolase [*Glycine max*]	KRH73948.1	INF	0.015
PREDICTED: magnesium-chelatase subunit ChlH, chloroplastic [*Elaeis guineensis*]	XP_010938532.1 (+1)	INF	0.015
hypothetical protein SOVF_064180 [*Spinacia oleracea*]	KNA19149.1	INF	0.015
unnamed protein product [*Coffea canephora*]	CDP04403.1	INF	0.028
60S ribosomal protein L26-1 [*Glycine soja*]	KHN08577.1 (+3)	INF	0.028
PREDICTED: LOW QUALITY PROTEIN: glyceraldehyde-3-phosphate dehydrogenase 2, cytosolic-like [*Malus domestica*]	XP_008353451.1	INF	0.028
chalcone synthase [*Citrus sinensis*]	ACB47461.1 (+35)	INF	0.028
PREDICTED: probable methyltransferase PMT20 [*Solanum lycopersicum*]	XP_004244424.1 (+4)	INF	0.028
PREDICTED: heme-binding-like protein At3g10130, chloroplastic [*Beta vulgaris* subsp. vulgaris]	XP_010675294.1 (+1)	INF	0.028
PREDICTED: delta-1-pyrroline-5-carboxylate synthase-like [*Sesamum indicum*]	XP_011076783.1 (+2)	INF	0.028
PREDICTED: 5-methyltetrahydropteroyltriglutamate--homocysteine methyltransferase-like [*Malus domestica*]	XP_008386852.1	INF	0.028
PREDICTED: 40S ribosomal protein S15 [*Sesamum indicum*]	XP_011091134.1	INF	0.028
BnaC04g47280D [*Brassica napus*]	CDX93168.1	INF	0.028
PREDICTED: 2-methylene-furan-3-one reductase-like isoform X1 [*Erythranthe guttata*]	XP_012855332.1 (+1)	INF	0.028
translationally controlled tumor protein-like protein [*Arabidopsis thaliana*]	AAM66134.1 (+30)	12.0	0.0049
ATP synthase beta subunit [*Kalanchoe daigremontiana*]	CAB89919.1	11.0	0.0082
PREDICTED: alpha-1,4 glucan phosphorylase L-2 isozyme, chloroplastic/amyloplastic-like [*Sesamum indicum*]	XP_011075582.1	10.0	0.014
ATP synthase CF1 alpha subunit (chloroplast) [*Euphorbia esula*]	AMC30641.1	9.0	0.023
unknown [*Glycine max*]	ACU19212.1 (+55)	9.0	0.023

ribulose-1,5-bisphosphate carboxylase/oxygenase large subunit, partial (chloroplast) [*Breweria rotundifolia*]	AAM55983.1	9.0	0.023
60S ribosomal L35-like protein [*Medicago truncatula*]	XP_003624180.1	9.0	0.023
unnamed protein product [*Coffea canephora*]	CDP06727.1 (+12)	9.0	0.023
Heat shock cognate 90 kDa [*Gossypium arboreum*]	KHG10495.1 (+3)	8.0	0.038
putative alcohol acyltransferase 2 [*Lavandula angustifolia*]	ABI48361.1 (+2)	8.0	0.038
PREDICTED: salicylic acid-binding protein 2-like= polyneuridine-aldehyde esterase [*Sesamum indicum*]	XP_011099227.1	8.0	0.038
heat shock protein 90-1 [*Nicotiana benthamiana*]	ALD51960.1 (+2)	8.0	0.038
hypothetical protein PHAVU_005G076400g [*Phaseolus vulgaris*]	XP_007149511.1	6.0	0.017
PREDICTED: patellin-3-like [*Sesamum indicum*]	XP_011096139.1	5.7	0.0049
granule-bound starch synthase 1, chloroplastic/amyloplastic, partial [*Genlisea aurea*]	EPS65732.1	5.5	0.027
PREDICTED: photosystem I reaction center subunit VI, chloroplastic-like [*Sesamum indicum*]	XP_011080763.1	5.3	0.0076
PREDICTED: tubulin beta-1 chain [*Sesamum indicum*]	XP_011074687.1	4.8	0.0016
PREDICTED: 60S ribosomal protein L7-2 [*Nelumbo nucifera*]	XP_010269530.1 (+1)	4.7	0.018
beta-tubulin, partial [*Genlisea aurea*]	EPS57653.1	4.4	0.00026
PREDICTED: probable aldo-keto reductase 2 [*Ricinus communis*]	XP_002518605.1	4.3	0.028
putative 1-deoxy-D-xylulose 5-phosphate reductoisomerase [*Hevea brasiliensis*]	AAS94121.1 (+17)	4.0	0.019
RNA-binding protein 3 [*Arabidopsis thaliana*]	AAA18380.1 (+1)	4.0	0.042
PREDICTED: phosphoglucomutase, cytoplasmic-like [*Nicotiana tomentosiformis*]	XP_009623305.1 (+2)	3.5	0.041
40S ribosomal protein S15-like protein [*Forsythia suspensa*]	ACI14379.1 (+10)	3.5	0.041
PREDICTED: chlorophyll a-b binding protein of LHCII type 1-like [*Vitis vinifera*]	XP_002273106.2	3.2	0.0025
unnamed protein product [*Coffea canephora*]	CDP12247.1	3.2	0.026
PREDICTED: ATP-dependent zinc metalloprotease FTSH 2, chloroplastic [*Sesamum indicum*]	XP_011082976.1	3.0	0.00025

PREDICTED: granule-bound starch synthase 1, chloroplastic/amyloplastic [*Sesamum indicum*]	XP_011081361.1	3.0	0.005
PREDICTED: dirigent protein 22-like [*Vitis vinifera*]	CBI30453.3 (+1)	2.9	0.0069
PREDICTED: coproporphyrinogen-III oxidase 1, chloroplastic [*Sesamum indicum*]	XP_011096874.1	2.9	0.0069
PREDICTED: glyceraldehyde-3-phosphate dehydrogenase B, chloroplastic [*Vitis vinifera*]	CAN69459.1 (+1)	2.9	0.033
S-adenosylmethionine synthetase 1 family protein [*Populus trichocarpa*]	XP_002320949.1	2.9	0.033
chloroplast 28 kDa ribonucleoprotein, partial [*Oryza sativa* Indica Group]	ABR25700.1 (+12)	2.7	0.028
PREDICTED: glucose-1-phosphate adenylyltransferase large subunit 3, chloroplastic/amyloplastic [*Sesamum indicum*]	XP_011090768.1	2.6	0.0033
ATP synthase CF0 subunit I [*Ruellia breedlovei*]	AJS14405.1	2.5	0.032
granule-bound starch synthase [*Ipomoea muelleri*]	AAD38466.1	2.5	0.032
PREDICTED: glutamate-1-semialdehyde 2,1-aminomutase 2, chloroplastic [*Jatropha curcas*]	XP_012090641.1	2.5	0.035
PREDICTED: LOW QUALITY PROTEIN: histone H4-like [*Malus domestica*]	XP_008359709.1	2.4	0.01
PREDICTED: probable carboxylesterase 8 [*Sesamum indicum*]	XP_011091180.1	2.4	0.047
4-hydroxy-3-methylbut-2-enyl diphosphate synthase [*Lavandula angustifolia*]	AGQ04156.1	2.4	0.047
PREDICTED: transketolase, chloroplastic [*Populus euphratica*]	XP_011034130.1	2.3	0.034
RecName: Full=Chlorophyll a-b binding protein AB80, chloroplastic; AltName: Full=LHCII type I CAB-AB80; Short=LHCP; Flags: Precursor	P07371.1 (+1)	2.2	0.0001 6
PREDICTED: tubulin alpha chain [*Erythranthe guttata*]	XP_012841365.1	2.2	0.019
vacuolar H+-pyrophosphatase [Oryza sativa (japonica cultivar-group)] [*Oryza sativa* Japonica Group]	BAA08232.1 (+29)	2.2	0.021
Superoxide dismutase [Cu-Zn], chloroplastic [*Gossypium arboreum*]	KHG11568.1 (+1)	2.2	0.04
granule-bound starch synthase 1 [*Nicotiana tabacum*]	AGH30272.1 (+1)	2.2	0.045
hypothetical protein M569_13065, partial [*Genlisea aurea*]	EPS61728.1	2.1	0.018

RecName: Full=Tubulin beta chain; AltName: Full=Beta-tubulin	P28551.2 (+6)	2.0	0.003
PREDICTED: ferredoxin-dependent glutamate synthase, chloroplastic [*Sesamum indicum*]	XP_011075414.1	2.0	0.024

Supplementary Table S3. Composition of tissues in leaf cross sections. Significance analyzed with Student's *t*-test.

	M area (%)	BS area (%)	M/BS area ratio	Epidermis area (%)	Vascular bundle area (%)	Air space area (%)
Terrestrial	30.2±2.8	7.0±1.3	4.5±0.6	27.2±3.6	2.8±1.0	33.1±5.6
Submerged	32.7±2.6	7.3±1.9	4.5±0.8	23.3±6.1	3.1±0.5	33.0±10.7
p vaule	0.25	0.63	0.94	0.40	0.72	0.99

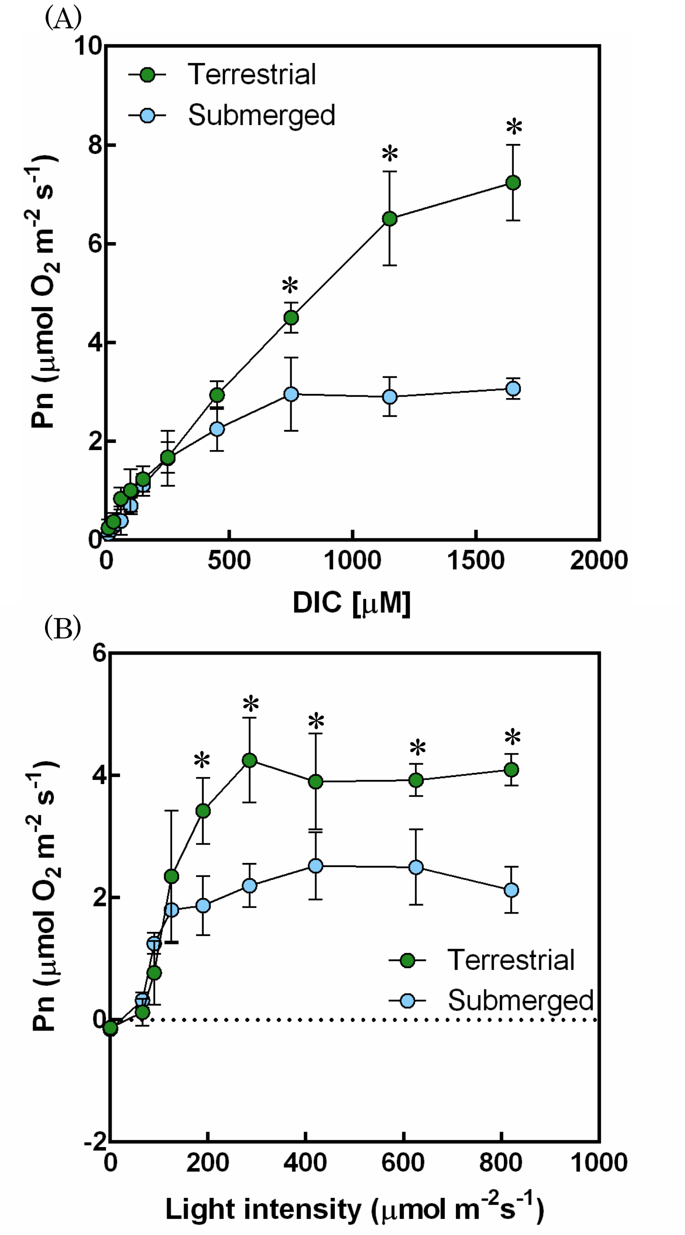

Supplementary Figure S1. The underwater photosynthetic rate per leaf area in terrestrial and submerged leaves of *H. polysperma* in response to DIC (**A**) and Light (**B**). (**A**) The underwater photosynthesis rate per leaf area in the terrestrial and submerged leaves of *H. polysperma* (n=3) in response to DIC. Light intensity and pH conditions were 285 μmol m^{-2} s^{-1} and at pH 6.3, respectively. (**B**) The underwater photosynthesis rate per leaf area in the terrestrial and submerged leaves of *H. polysperma* (n=3) in response to light intensity. Results in panels (**A**) and (**B**) are expressed as means ± SD. Significance was analyzed by two–way ANOVA with Hlom–Sidak test ($*p < 0.05$).

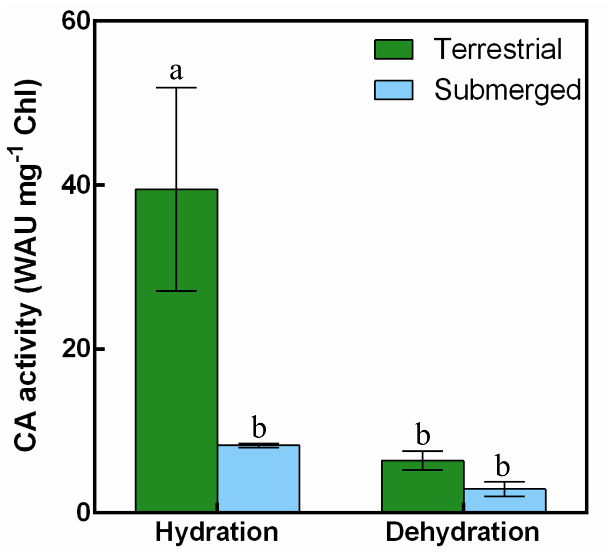

Supplementary Figure S2. CO_2 hydration and HCO_3^- dehydration reactions of CA activity in terrestrial and submerged leaves. Green and blue bars indicate the CA activity of terrestrial and submerged leaves, respectively. Results are expressed as the means ± SD (n = 4). Data were analyzed with Tukey's HSD test. Different letters indicate statistical differences between the enzymatic activities (p<0.05).
